# Extension of Life Span by Impaired Glucose Metabolism in *Caenorhabditis elegans* Is Accompanied by Structural Rearrangements of the Transcriptomic Network

**DOI:** 10.1371/journal.pone.0077776

**Published:** 2013-10-30

**Authors:** Steffen Priebe, Uwe Menzel, Kim Zarse, Marco Groth, Matthias Platzer, Michael Ristow, Reinhard Guthke

**Affiliations:** 1 Systems Biology and Bioinformatics Group, Leibniz Institute for Natural Product Research and Infection Biology - Hans-Knoell-Institute, Jena, Germany; 2 Department of Human Nutrition, Institute of Nutrition, University of Jena, Jena, Germany; 3 Genome Analysis Group, Leibniz Institute for Age Research - Fritz-Lipmann-Institute, Jena, Germany; University of Erlangen-Nuremberg, Germany

## Abstract

Glucose restriction mimicked by feeding the roundworm *Caenorhabditis elegans* with 2-deoxy-D-glucose (DOG) - a glucose molecule that lacks the ability to undergo glycolysis - has been found to increase the life span of the nematodes considerably. To facilitate understanding of the molecular mechanisms behind this life extension, we analyzed transcriptomes of DOG-treated and untreated roundworms obtained by RNA-seq at different ages. We found that, depending on age, DOG changes the magnitude of the expression values of about 2 to 24 percent of the genes significantly, although our results reveal that the gross changes introduced by DOG are small compared to the age-induced changes. We found that 27 genes are constantly either up- or down-regulated by DOG over the whole life span, among them several members of the cytochrome P450 family. The monotonic change with age of the temporal expression patterns of the genes was investigated, leading to the result that 21 genes reverse their monotonic behaviour under impaired glycolysis. Put simply, the DOG-treatment reduces the gross transcriptional activity but increases the interconnectedness of gene expression. However, a detailed analysis of network parameters discloses that the introduced changes differ remarkably between individual signalling pathways. We found a reorganization of the hubs of the mTOR pathway when standard diet is replaced by DOG feeding. By constructing correlation based difference networks, we identified those signalling pathways that are most vigorously changed by impaired glycolysis. Taken together, we have found a number of genes and pathways that are potentially involved in the DOG-driven extension of life span of *C. elegans*. Furthermore, our results demonstrate how the network structure of ageing-relevant signalling pathways is reorganised under impaired glycolysis.

## Introduction

The nematode *Caenorhabditis elegans* has emerged as an excellent model for the study of the mechanisms of ageing and determination of longevity [1]. Beside other interventions, reduced nutritional uptake, often termed calorie restriction (CR) or dietary restriction (DR) is affecting the life span of *C. elegans* and a multitude of other eukaryotes, including mammals [2], [3]. *C. elegans* fed with a chemical inhibitor of glycolysis, namely 2-deoxy-D-glucose (DOG), exhibit considerably extended life span. DOG, in contradiction to D-glucose, cannot be metabolized in the glycolytic pathway. This results in the fact that less glucose is available for ATP production, and thus makes DOG-feeding of the roundworms equivalent to glucose restriction. The life span extending effect of this treatment was shown to be mediated by impairment of the insulin/IGF1 signalling pathway [4].

In a recent paper, Zarse et al. [5] show that the life span extension in *C. elegans* induced by impairment of insulin/IGF1 signalling is connected with remarkable changes on the metabolic level. It was revealed that reduced insulin/IGF1 signalling initially triggers a shift of the mitochondrial metabolic regime towards oxidative proline metabolism. The metabolic shift is connected with an intermittent flash of reactive oxygen species (ROS), which in turn provokes a stress response program of the cell, eventually leading to a permanently elevated antioxidant defense, attestable by increased levels of superoxide dismutase (SOD) and catalase (CTL). This adaptive response has emerged as a key mechanism of stress resistance and elongated life span, and has been established in the literature with the designation hormesis or “mitohormesis” [6].

In this paper, we aim at facilitating further understanding of the molecular mechanisms behind the DOG-driven life extension of the roundworm by investigating age effects on the transcriptomic level. Transcriptome data at different ages, measured by RNA-seq, for worms exposed to DOG and for controls were analyzed. We conducted a comprehensive bioinformatic survey of the data, in order to elucidate alterations on the transcriptome level in the course of ageing. By taking into account four age levels (1 day, 5 days, 10 days, and 20 days) covering the whole lifespan of wildtype roundworms, the effects of the DOG-treatment were analyzed and compared to expression changes caused by unperturbed ageing.

This work covers several levels of gene expression analysis. By the analysis of sample correlation, global effects of both ageing and DOG-treatment have been investigated, while identification of differential expression at single age levels allows quantifying the sole influence of DOG on gene expression. Clustering based methods were used in order to find groups of genes mostly effected by the treatment with DOG. However, only looking at absolute changes of expression levels of sets of genes is certainly not sufficient in order to understand the changes on the transcriptome introduced by the process of ageing, effecting a multitude of pathways [7]. In the recent years, evidence has accumulated that life span is considerably influenced by the regulation of the complex interplay between cellular components like transcripts, proteins, and metabolites [8]. A central age-determining role is associated with the mTOR signalling pathway. It has been shown in several model organisms that inhibition of this pathway extends lifespan and protects against age-related impairment of cellular function [9], [10]. As a matter of course, such changes are not manifested in the genomic set-up of a given individual. For this reason, we conducted an investigation of deregulation of several pathways as a consequence of impaired glycolysis. We applied two different network approaches which focus on interactions between multiple genes. We created correlation based networks for normal and perturbed ageing, similar to the analysis of co-expression difference networks across different tissues introduced by Southworth et al. [11]. Looking at the differences of co-expression between two networks enables a genome-wide view as well as insight into the interaction of genes in particular pathways. In addition to correlation based difference networks, we created mutual information (MI) networks for selected pathways. MI measures the degree of statistical dependency and the nonlinear relationship between the expression levels of genes and has been successfully applied as co-expression measure for gene expression data [12], [13]. Again, separate networks can be created for the glucose restricted condition and for the controls by estimating the pairwise MI across gene expression during ageing. Afterwards, estimation of typical network measures like the distribution of node degrees allows us to compare and characterize these pathways and the changes introduced by the DOG-treatment to the different complex networks.

Our work revealed that genes of the electron transport chain are strongly effected by states of impaired glycolysis. Genes of this pathway have been previously found to be age regulated across different species [14]. In addition to effects on single pathways, we also found a widespread structural rearrangement of the transcriptional co-expression network.

## Results

### Impact of Impaired Glycolysis on the Overall Transcription Pattern is Small Compared to Impact of Ageing

**Figure 1:**
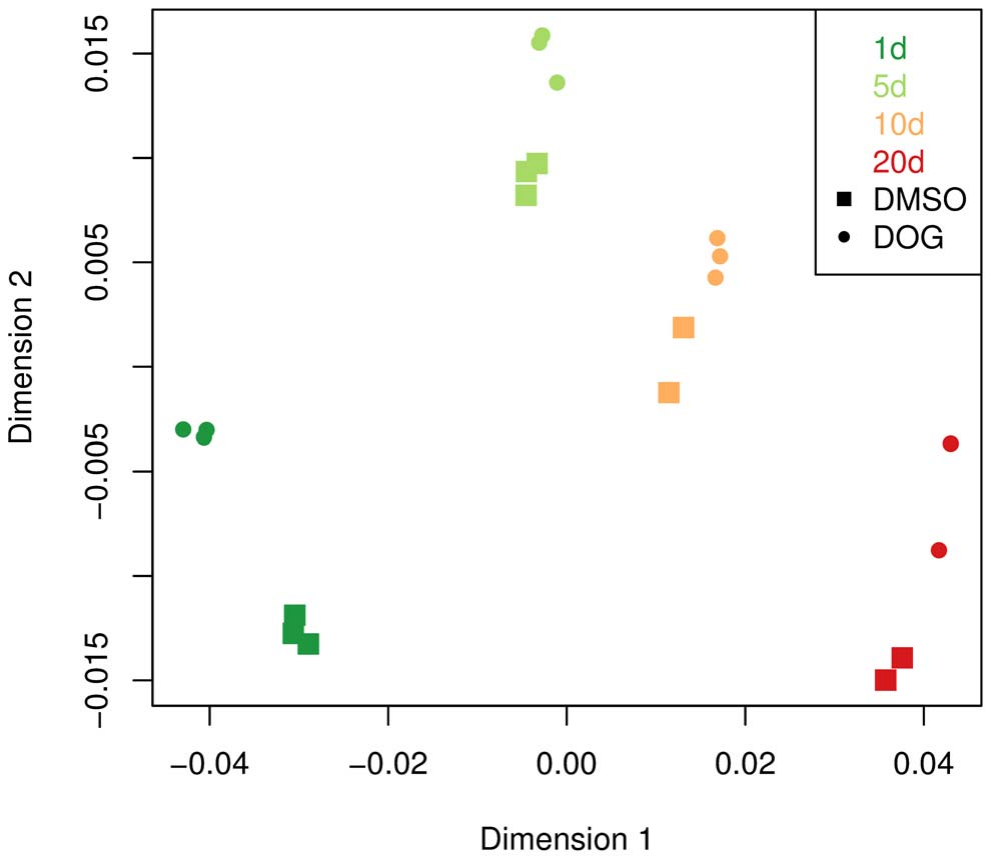
Multi-dimensional scaling plot for 21 samples. The MDS was performed using the log2 RPKM values and is based on Pearson correlation between the expression profiles of all samples. Samples are primarily arranged according to age (indicated by color), and only then according to treatment (indicated by shape).

First, we computed the Pearson correlation between all pairs of samples and visualized the resulting correlation values by multi-dimensional scaling (MDS, Figure 1). The plot reveals that it is possible to distinguish three scales of dissimilarity between the samples, which refer, ranked by their impact, a) to age, b) to treatment, and c) to repeated measurement of samples with the same age and treatment. The distances are biggest between samples of different age, leading to the situation that the four age groups form their own clusters, with a continuous progression of these clusters from the left bottom of the plot (1-day old worms) via the top to the right bottom of the plot (20-day). Thus, the changes introduced to the shape of the overall expression profile by the ageing process exceed changes caused by the DOG-treatment - impaired glycolysis apparently changes these profiles only selectively. Otherwise, the plot reveals that DOG introduces reproducible changes to these profiles. The latter becomes apparent through the fact that labels referring to treated samples clearly separate from labels standing for untreated ones within each age cluster or, with other words, changes introduced by impaired glycolysis exceed the variation of the correlation coefficient within replicates.

### The Fraction of Down-regulated Genes in DOG-treated Worms Increases with Age

**Figure 2:**
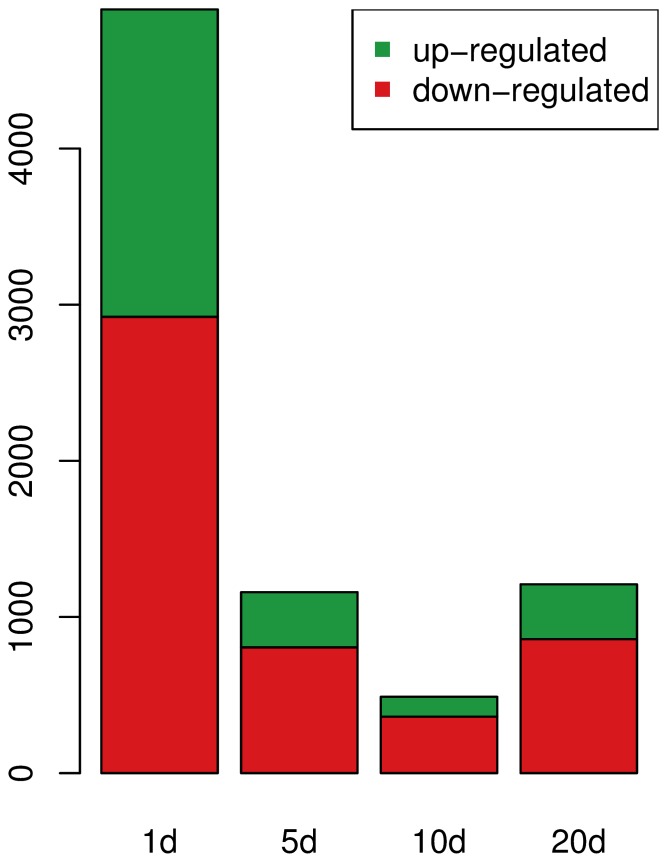
Number of differentially expressed genes (DEG) between DOG-treated worms and controls. While the total number of DEG decreases from 1-day old worms to 10-day old worms, the fraction of down-regulated genes increase with age under impaired glycolysis.

The absolute numbers of differentially expressed genes (DEG) between DOG-treated vs. control samples for different age levels are depicted in Figure 2 and listed in Table 1 (see Table 11 for a complete list of DEG). At the age of 1 day, 4,891 genes are differentially expressed between the two conditions. The number of DEG at later stages is considerably smaller (5 d: 1,159, 10 d: 489, 20 d: 1,209). The majority of DEG is down-regulated at all stages (1 d: 59.7% 5 d: 69.5%, 10 d: 74%, 20 d: 70,9%). This indicates an overall reduction of transcriptional activity induced by DOG.

**Table 1:** Number of differentially expressed genes between DOG-treatment and controls.

	DEG	up-regulated	down-regulated
1 day	4,891	1,969	2,922 (59.7%)
5 day	1,159	353	806 (69.5%)
10 day	489	127	362 (74%)
20 day	1,209	351	858 (70.9%)

The first column reports the total number of differentially expressed genes, the 2nd column shows the number of up-regulated genes under DOG-treatment compared to controls, and the 3rd column shows the number of down-regulated genes under DOG-treatment compared to controls.

Comparable results have been found in *C. elegans* during the exposure to an inhibitor of mitochondrial complex I, namely rotenone, where the maximum number of DEG was observed already after 24 h of treatment, and then decreases for all later time points [15].

**Figure 3:**
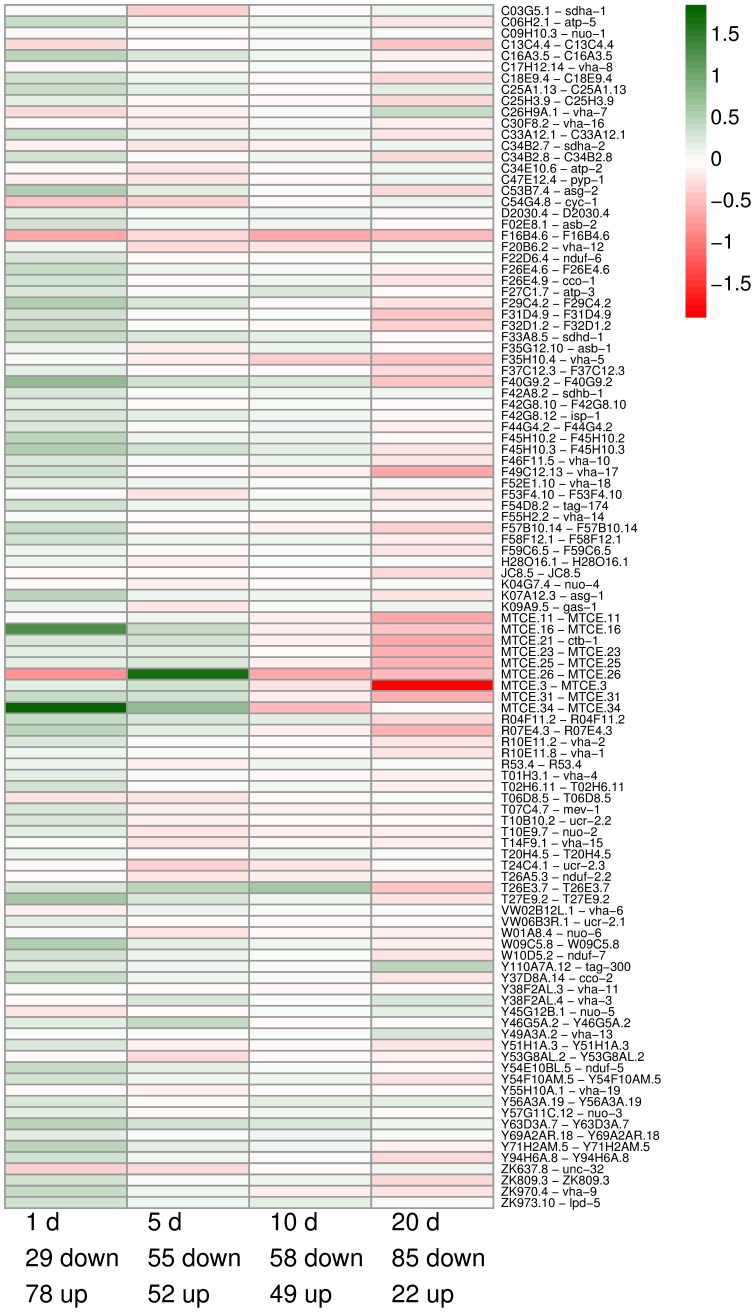
Change of expression levels of 107 oxidative phosphorylation pathway genes during DOG-treatment. The heatmap shows the log2-foldchange for the expression levels of DOG-treated worms and controls. Green color indicates an up-regulation by DOG-treatment, red color indicates a down-regulation by DOG-treatment, compared to controls. A majority of these genes is down-regulated with age by the DOG-treatment. Note especially the strong regulation of mitochondrially encoded genes (starting with MTCE).

The oxidative phosphorylation (“Oxphos”) pathway plays a key role for the life span extending effect of impaired glycolysis in *C. elegans*. In a recent paper, Schulz et al. [4] have shown that impaired glycolysis in the roundworms causes an activation of this pathway, followed by a temporarily increased production of reactive oxygen species (ROS). This, in turn, stimulates the establishment of a molecular defense mechanism against ROS, thereby increasing life span. Indeed, looking at the change of the expression levels of the Oxphos genes brought about by the DOG-treatment, we see an up-regulation of the majority of the Oxphos genes compared to controls at the first day (Figure 3). However, with increasing age, the fraction of up-regulated genes in the Oxphos pathway decreases, and at very old age (20 days), the Oxphos genes of the DOG-treated worms are mostly down-regulated when compared to controls.

Four genes are constantly significantly up-regulated, while eight genes are constantly down-regulated under DOG at all four age levels (Table S2). Among these genes *cyp-35A5* is up-regulated at all four age levels, while *cyp-13A5* is constantly down-regulated. Both genes are members of the cytochrome P450 family and have been associated with ageing previously. Furthermore, it is known that members of the *cyp-35x* family are up-regulated in response to xenobiotics [16].

### Expression of Antioxidant Enzymes is Inconsistent during DOG-treatment

**Figure 4:**
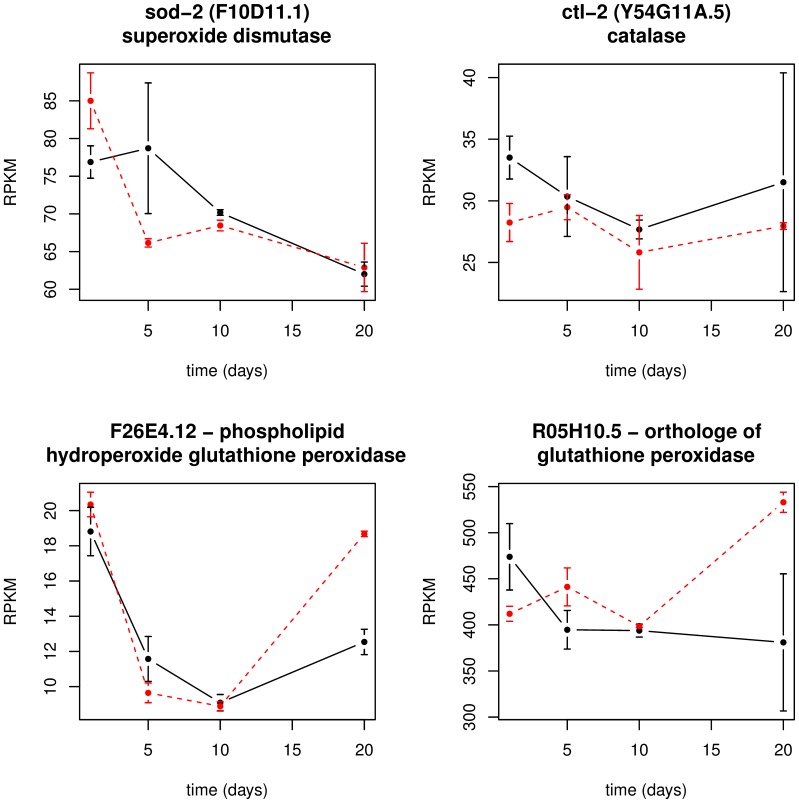
The temporal expression profiles of four genes encoding antioxidant enzymes. The plot shows the mean RPKM values as well as the standard deviation versus age in days for superoxide dismutase *sod-2*, the peroxisomal catalase *ctl-2* and two genes encoding glutathione peroxidases. Black curve: controls; red curve: DOG-treatment.

We compared the temporal expression profiles of genes encoding the antioxidant enzymes superoxide dismutase (*sod-2*), catalase (*ctl-2*), and glutathione peroxidase in the DOG-treated worms and in the controls. The *sod-2* gene is the major manganese superoxide dismutase in the mitochondria. Therefore, if life span of the worms is (partly) determined by the efficiency of ROS scavenging, and if the life span extension by DOG-treatment functions via improved removal of ROS, we might suppose an influence of the treatment on the expression profile of this gene. Surprisingly, the differences of *sod-2* expression between the DMSO- and the DOG-case are not statistically significant and are restricted to the first and second age levels (Figure 4). It was reported that suppression of the *ctl-2* gene shortens life span in *C. elegans* [17], though we see a slight down-regulation of the gene over the whole life span under DOG-treatment. In contrast, the two *C. elegans* genes which supposably encode glutathione peroxidases in (*F26E4.12*, *R05H10.5*), are clearly up-regulated compared to the controls at old age (20 days) in the DOG-treated worms (Figure 4 and S1 for all genes encoding antioxidant enzymes). Investigation of the glutathione metabolism pathway (KEGG-ID cel00480) reveals that a few genes with detoxifying function (*gst4, gst38, gst30, gst5, gst8*) are up-regulated in the DOG-treated worms, while most of the remaining genes of this pathway are down-regulated (Figure S2).

### Ageing-relevant Genes Display Opposite Monotonic Profiles during DOG-treatment

Because ageing is a continuous process, it can be hypothesized that genes possessing key relevance for ageing (or genes that can be seen as markers for age) change their expression values monotonically over time, while genes with irregular temporal expression patterns might be associated with response to environmental conditions, with the circadian rhythm or other processes. In order to identify genes that change their expression levels monotonically with age, we calculated the Spearman correlation coefficient of each gene’s temporal profile with a linearly increasing sequence. Replicates for each time point were incorporated by a random sampling approach. Subsequently, we classified genes into three classes according to their behaviour with age: a) non-uniformly regulated genes, b) monotonically up-regulated genes, and c) monotonically down-regulated genes (Table 2).

**Table 2:** Number of monotonically up- and down-regulated genes.

class	DMSO	DOG
monotonically up-regulated	2,473	4,080
monotonically down-regulated	1,589	2,189
non-uniformly	12,151	9,949

The number of genes whose expression values are monotonically up- and down-regulated, respectively, for the DOG-treated worms and for the controls. Note, that monotonic behaviour does not necessarily include differential expression.

Next, we searched for genes that reversed their monotonic behaviour of expression under DOG-treatment. We found 9 genes that were monotonically up-regulated with age in controls but monotonically down-regulated with age under DOG, and 12 genes that changed from down- to up-regulated (Table S3). Amongst the 9 genes changing from up- to down-regulated, we find genes coding the ubiquitin conjugating enzyme (*ubc-7*) and the antioxidant enzyme peroxiredoxin (*prdx-2*), known to be relevant for life span [18]. The *daf-21* and *C30C11.4* genes, members of the heat shock protein Hsp90 and Hsp70 family, respectively, changed from down- to up-regulated. Loss of *C30C11.4* has been reported to result in reduction of adult life span in otherwise longlived animals [19]. Several of the herewith identified genes have not been linked previously to ageing related processes in *C. elegans*.

### Ageing-relevant Genes show Significant Negative Correlation between Controls and DOG Treated Samples

We used a permutation approach to identify 246 genes whose transcript levels are significantly negatively correlated over age between controls and DOG-treated worms. Such genes exhibit opposite trends with age under the different metabolic regimes, and therefore earn special attention. Subsequently, they were clustered according to their temporal profiles using a fuzzy c-means algorithm. The optimum number of clusters was determined to be 9, using a combination of several cluster validation indexes [20]. The resulting clustering profiles showed clear opposite behaviour between DOG-treatment and controls (Figure S3), while more genes were down-regulated with age under DOG-treatment (154 genes, clusters 2, 4, 5, 6 & 7). Note that the results presented here are similar to those obtained in the previous section, with the distinction that we did not require monotony here. Only 22 genes were found as up-regulated with age under DOG-treatment (cluster 3) and the remaining 3 cluster profiles displayed irregular but likewise opposite temporal behaviour between treatments and controls.

We used the DAVID bioinformatics resource [21] to determine significantly enriched functional categories using the “Functional Annotation Clustering” tool for the clusters identified previously (Table S4). Again, special attention was directed to the clusters containing genes whose temporal transcript profiles displayed opposite quantitative outcome between untreated and DOG-treated worms. The 154 genes down-regulated with age under DOG-treatment are, among others, enriched with the GO terms “cell redox homeostasis” (GO:0045454), “ubiquinone biosynthetic process” (GO:0006744), “determination of adult life span” (GO:0007568), and “RNA polymerase activity” (GO:0034062). The 22 genes up-regulated with age under DOG-treatment are significantly enriched () with GO categories related to growth and development, like “growth” (GO:0040007), “regulation of growth rate” (GO:0040009), and “positive regulation of multi-cellular organism growth” (GO:0040018).

### Global Co-expression of DOG-treated Worms is more Dense when Compared to Controls

In order to investigate co-expression patterns of genes under a particular condition, we constructed correlation networks based on the transcriptome data. To start with, we investigated untreated and treated worms separately, i.e. we constructed two networks: one for control worms (DMSO-network) and another one for DOG-treated worms (DOG-network). In these networks, genes are represented by nodes, and the Pearson product moment correlation coefficient between the temporal transcript profiles of a given gene pair was used as weight for the edge connecting this gene pair in the network. The significance of correlation between any pair of genes was estimated and the replicates were included by randomly sampling and subsequent averaging. Genes were only connected in the network when the computed p-value was .

Remarkably, the total number of co-expressed genes and the number of edges are bigger in the DOG-network (Table 3), supporting the hypothesis that impaired glycolysis on average increases the global interconnectedness of the genes. This is also supported by the higher values for mean and maximum node degree displayed by the DOG-network. However, as shown below, this is not necessarily true for every individual pathway.

**Table 3:** Key parameters of the two correlation networks.

network parameter	DMSO	DOG
number of nodes	6,273	9,287
number of edges	711,093	1,313,511
mean node degree	96.67	178.56
maximum node degree	1,124	1,398
gamma exponent	−0.74	−0.85

Characteristics of the networks constructed for controls and worms under DOG-treatment. The gamma-parameter (last row) refers to the exponent of the power law usually assumed for the frequency of the node degree.

We investigated the frequency distribution of the number of edges for the nodes in the network (node degree) for both the DMSO- and the DOG-case. As expected, we have a large number of genes with relatively low node degree, and fewer genes with high node degree, so called hubs. We fitted the frequency distribution of the node degree to a power law in the form , where is the node degree, denotes the frequency of occurrence of this node degree in the network, and and are constants to be fitted. The parameter describes how fast the frequency falls with increasing node degree, while is a scaling constant. The node degree distribution of many biological networks (asymptotically) follows such a power law, according to the concept of scale-free networks [22]. As seen in Table 3, the values of for both networks are about 0.8, the value for the DOG-network being slightly bigger.

### Ageing-relevant Pathways are among the most Strongly Affected by DOG Feeding

**Figure 5:**
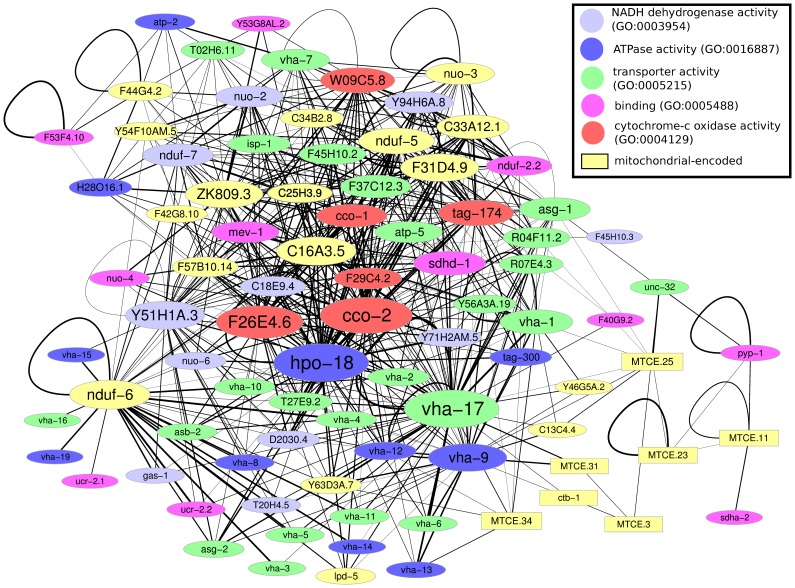
Correlation difference network for genes of the oxidative phosphorylation pathway. Only edges with a weight were drawn. All black edges represent positive weights, i.e. all shown genes exhibit increased co-expression under DOG-treatment. The line width denotes the extent of correlation difference between DOG and DMSO. Several genes present a hub-like structure with a maximum node-degree of 57 for *hpo-18* and *vha-17*. Mitochondrially encoded genes are represented by rectangular shaped nodes, which show less increased co-expression. The node color denotes the assignment to selected GO categories (see legend).

Investigating differences of co-expression patterns between two networks permits a more universal picture of gene expression changes between two conditions, in contrast to a single-gene comparison. In order to visualize effects of impaired glycolysis on the co-expression networks, we constructed difference networks (DN) by subtracting the corresponding weights of the DMSO- from those of the DOG-network. DN have already been successfully applied in order to investigate global changes in gene expression between young and old mice [11]. In our difference network, the weights between gene and gene were calculated as , which means that we do not distinguish between positive or negative correlation, but focus on absolute correlation changes. In Figure 5, we show a part of the difference network obtained for the genes of the Oxphos KEGG-pathway. As apparent in the figure, all edge weights are positive, i.e. all genes of the Oxphos pathway increased their correlation under impaired glycolysis. Taken together, the difference network based on correlation reveals that co-expression in the Oxphos pathway becomes tighter when impaired glycolysis applies.

Next, we determined the KEGG signalling pathways and GO-terms that are mostly affected by the impaired glycolysis by investigation of the respective sub-networks of the complete difference network. Pathways or GO-terms with disproportionately many connected genes in the corresponding difference network are those whose co-expression is mostly affected by impaired glycolysis. Table S5 lists the most significantly connected pathways/sub-networks. As a main result, we find that among the KEGG-pathways most vigorously changed by impaired glycolysis are “Ribosome”, “Metabolic pathways” and Oxphos. A large set of significantly connected GO-categories have been found, including e.g. “regulation of growth” (GO:0040008) or “aging” (GO:0007568).

### Impaired Glycolysis Causes Structural Re-organisation of Ageing-relevant Signalling Pathways

**Figure 6:**
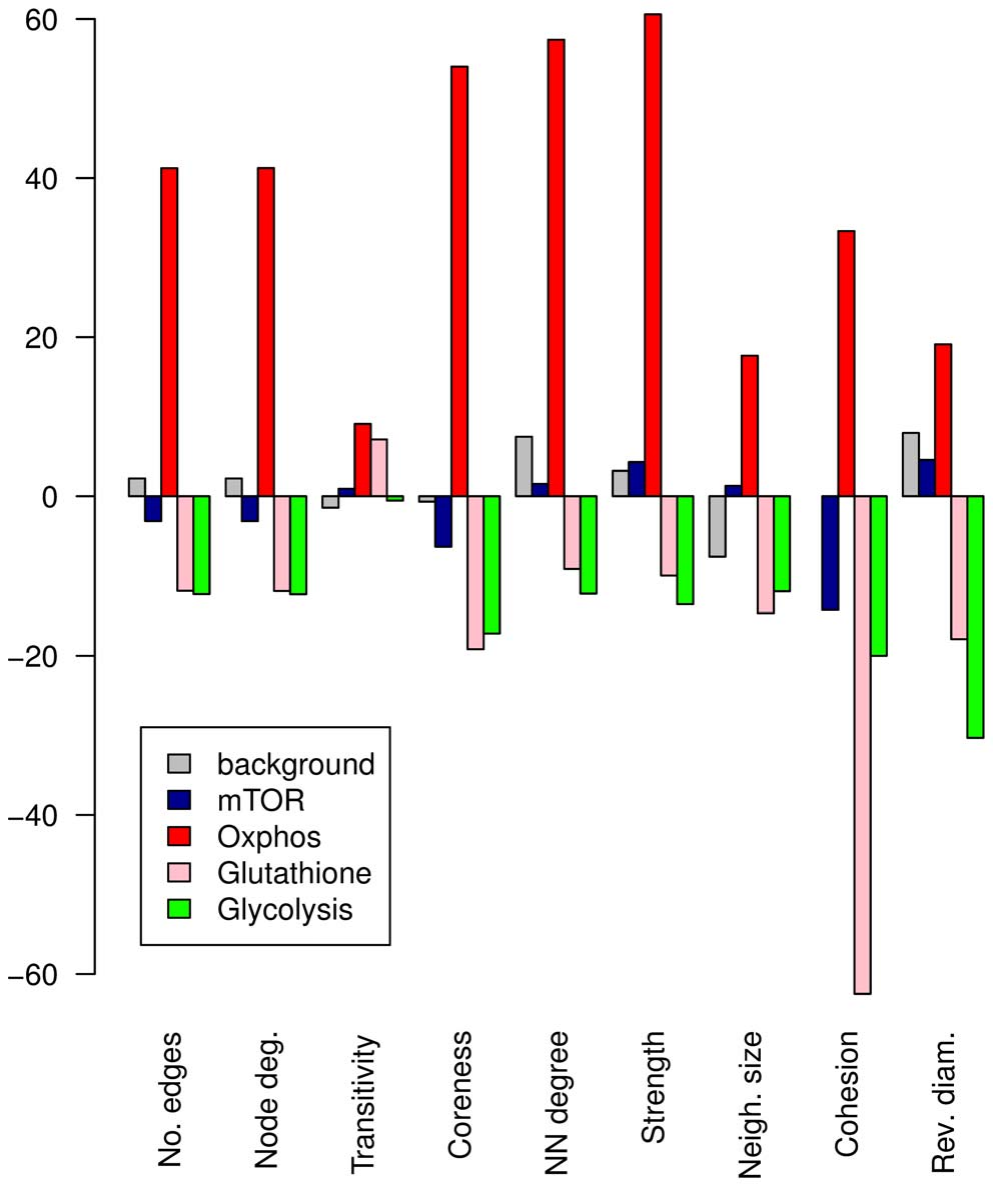
Graphical representation of the percentage change of characteristic MI-network parameters. The x-axis shows nine network parameters characterizing the network structure. The ordinate shows the percentage change of the corresponding parameter when DOG feeding replaces standard diet. Note that we plot the percentage change of the reciprocal value of the network diameter, thereby ensuring that upward pointing bars consistently indicate better connectivity under DOG-treatment (compared to controls), and downward-pointing bars indicate relaxed network connectivity in the DOG-case. The colors of the bars indicate the gene set the change was recorded for: grey bars - randomly chosen “background” genes; blue bars - mTOR pathway genes; red bars - oxidative phosphorylation pathway; red bars - glutathione metabolism pathway; green bars - glycolysis pathway.

We calculated the mutual information (MI) matrices based on the gene expression profiles for the glycolysis pathway and for other pathways known to be ageing-relevant, both for the DOG-treatment and the controls. In order to be able to compare the obtained results, the networks were also constructed for a background gene set (Table 4). Next, we calculated a number of network characteristics in order to uncover differences that emerge when the worms are kept under glucose-restricted conditions. In Figure 6, we show the percentage change of this network parameters for the individual pathways. The complete results of the calculations are reported in Table S6. Because diameter is the only parameter that indicates a tighter network when getting smaller, we show the percentage change of the reciprocal value of the diameter (i.e. 1/diameter) in the bar-plot (see Materials and Methods for details of selected parameters). Hence, bars pointing upwards consistently indicate a tighter network under DOG compared to controls, and bars pointing downwards indicate that the network is relaxed under DOG-treatment. Figure 6 discloses that the introduced changes differ remarkably between the individual pathways. While the background genes and the mTOR pathway genes show merely small percentage changes with both elevated and reduced parameter values, the Oxphos pathway data reveal a distinct enhancement of co-expression under DOG-treatment. This observation is consistent with earlier findings across different species [23], and with findings obtained by the correlation based networks. The higher interconnectedness of genes in the Oxphos pathway suggests a changed regime of mitochondrial activity in the worms held under glucose restriction conditions. In contrast, the connectivity of the glycolysis pathway decreases under DOG diet, pointing to a loosening of co-expression in this pathway.

**Table 4:** Selected gene-sets for mutual information networks.

pathway	KEGG-ID	number of genes
Background	–	1,000
mTOR pathway	cel04150	25
Oxidative phosphorylation	cel00190	110
Glutathione metabolism	cel00480	37
Glycolysis pathway	cel00010	40

Mutual information networks were created for these selected KEGG pathways and for both conditions. Afterwards, characteristic network parameters were compared in order to evaluate the influence of DOG-treatment on these pathways.

As stated above, the mTOR pathway genes display only small changes of the average network parameters. However, we discovered a structural re-organization of co-expression in this pathway that does not occur in the other pathways investigated in this study. For all the gene sets mentioned in Table 4, we also determined the hubs, i.e. genes in the network which have significantly more connections to their neighbors than the rest of the genes. In order to provide a quantitative measure of how strongly these genes are connected in both the control and the DOG-case, we estimated the graph strength. The graph strength of a node in a network is the sum of the weights of all edges connected to this node. We found that the administration of DOG changes the set of hubs in the mTOR pathway: three genes (*rps-6, let-363, mpk-1*) become hubs under DOG-treatment, while two other genes (*mop-25.1, ife-2*) lose their hub status when standard diet is replaced by DOG-feeding (Figure S4 and Table S6).

Most of the genes changing hub status under DOG-treatment have earlier been associated with effects on life span in *C. elegans*. The *let-363* gene is orthologous to the human *FRAP1* gene, the actual mTOR gene, and is classified in the Gene Ontology (GO) as determining adult life span (GO:0008340). The MAP kinase *mpk-1* - the ortholog of the human *ERK* gene - is a major communicator of cellular responses to external signals, and is involved in the regulation of cell survival and apoptosis. Loss of *rps-6* activity in adult animals extends life span [24]. Although the connectivity of this gene is increased under DOG-treatment, the relative levels of transcription are decreased at old age when DOG is administrated (Figure S5). The *ife-2* gene (ortholog of human *EIF4E*) encodes the translation initiation factor 4F - loss of this gene in adult animals extends life span [25].

## Discussion

Here we present different approaches for the comparison of time resolved gene expression data - measured by RNA-seq - across the complete life span of *C. elegans* under two different conditions. We passed through different levels of expression analysis, starting from the complete set of annotated and expressed genes, proceeding to different gene subsets and functional categories. Beyond the usage of already established and conventional methods, like differential expression and correlation analysis, we also applied new methods suited for non-normally distributed expression data (e.g. the permutation based test applied in order to find genes with opposite temporal behaviour between the two conditions).

To start with, the investigation of the global expression profiles revealed that the effects of ageing are stronger compared to the effects introduced by treatment with DOG (Figure 1). However, the changes on the overall expression caused by the DOG-treatment clearly exceed the variation within the replicates. Taking into account the fact that DOG-treated worms have a longer life expectancy, we can conclude that it seems to be sufficient to make selective changes to the expression patterns in order to prolongate life. We show below that DOG indeed introduces specific changes to the interaction between particular genes and gene groups, and that it is not necessary to reconfigure the whole transcriptome in order to make the worms longer-lived.

Following the disposable soma hypothesis [26], we might expect a more pronounced alteration of the transcriptome after the end of fertility, i.e. between the age of 10 and 20 days. However, at least on the basis of overall sample correlation, we cannot observe such an accelerated change, but the clusters corresponding to a particular age seem to relocate continuously. We have to take into account that the transcriptome data were not taken at equidistant age levels but differ by 4, 5, and 10 days, respectively. Considering that, the estimated changes per day are biggest between day 1 and day 5, while the 5-day to 10-day and the 10-day to 20-day changes are rather moderate and occur with an approximately equal speed (we can guess that the distance between 10 and 20 days is twice that between 5 and 10 days but have to take into account that also the double amount of time has gone).

The latter appears true for controls as well as for DOG-treated samples, a situation that seems to be contradicting the prolonged life of the DOG treated worms [4]. We could naively expect that older DOG samples should be located somewhat closer to younger controls if DOG would just delay the ageing-related changes on the level of global transcription patterns. However, this is not the case here, again speaking for the selectivity of the modifications that lead to a prolongated life.

It speaks for the quality of the measurements that replicates - i.e. samples of same age and treatment - are located closely together in the MDS-plot and thus, reveal the largest correlation (Figure 1). This circumstance can be used for validation of the data. Respectively, one sample was excluded from the analysis because of its high variation and a lack of concordance of its RPKM values with the other replicates in this age group. In the MDS-plot, this sample clearly appeared as an outlier before it was removed from the data set (data not shown).

When following the above arguments, we should keep in mind that the MDS-plot based on Pearson correlation can only give us a gross view on the alterations caused by the administration of DOG. In particular, by calculating the correlation between samples we are only able to detect changes of the shape of the overall expression profiles, but we do not consider possible changes of the magnitude of expression following DOG-treatment. Hence, more subtle changes were subsequently investigated.

Although DOG is administrated over the whole life span of the worms, the number of DEG between DOG-treated worms and controls declines from the age of one day to the age of 10 days (Figure 2). Only at very old age (20 days), this number increases again. We can hypothesize that the significant initial change of expression strength of more than 4,800 genes (Table 1), constituting about 24% of all protein-coding genes, is connected with the initiation of a hormesis effect taking place, as described in the papers by Schulz and Zarse et al. [4], [5]. They show that impaired glycolysis primarily provokes dramatic changes on the molecular level, connected with a transiently increased production of reactive oxygen species (ROS). This, in turn, causes a long-term re-programming of the cell’s metabolic regime, leading to the establishment of stable, long-term persisting mechanisms of antioxidant defense.

In this perspective, the strong initial transcriptional changes might be attributed to the temporary molecular reaction to the ROS flash the cells are exposed to. In later life, the changes brought about by the ROS flash are piece by piece reversed to return the system to an age-specific steady state, while a subset of processes, e.g. the antioxidant defense loops, remain on a deflected level over a considerable part of the life span. The long-lasting effects of a transient ROS pulse are confirmed by measurements showing that already a short-term treatment with DOG extends life span by approximately the same amount as a life-long treatment [4]. A similar effect can be observed in *C. elegans* during the exposure to rotenone, an inhibitor of mitochondrial complex I. It has been shown that already an exposure to rotenone for 5 days was sufficient to extend life span [15].

Because the dosage of DOG is kept constant over the whole life span, there is no straightforward explanation for the rise of the number of DEG near the end of the worm’s lifetime, at an age of 20 days. It is though possible that this final increase of the differences in gene regulation between treated worms and controls is a consequence of the decline of the homeostatic capacity with age, which may be more pronounced in the controls with their shorter life expectancy. The latter is supported by the experimental finding that the survival curves of treated and untreated worms start to differ at the age of approximately 20 days [4].

While the number of down-regulated genes under DOG-treatment is consistently higher than the number of up-regulated genes, this effect becomes even more pronounced with progressing age. Thus, under the influence of impaired glycolysis, the transcriptional machinery is - on average - turned down, something that might be a measure to spare resources in the absence of high-energetic digestible glucose.

It is still under debate to what extend the free radical theory of ageing applies [17], [27]. The basic proposition of this theory is that ageing is caused by the accumulation of damage induced by the detrimental effects of ROS. ROS are unavoidable by-products of the processes taking place in the electron transport chain of the mitochondria [28], [29]. Accordingly, it should be possible to reduce the destructive impact of ROS and to prolongate life by reducing the turnover of mitochondrial energy production, i.e. by down-regulating the mitochondrial metabolism.

While this theory was widely accepted for a long time, conflicting hypothesis have emerged in the recent years. It was experimentally proven that life extension by impaired glycolysis can go along with an increased oxygen consumption in *C. elegans*, when the worms compensate for impaired levels of glycolytic ATP by accelerated production of mitochondrial ATP, the latter requiring gaseous oxygen [4], [5]. The life extension can then be explained by the hormesis effect already mentioned above.

Transcript levels of genes encoding the Oxphos pathway, in glycolytically impaired worms compared to controls, (Figure 3), can give a clue about the changed intensity of mitochondrial energy production under DOG-treatment. It emerges that the expression of the majority of the genes belonging to the Oxphos pathway is increased in DOG-treated worms at the age of one day, suggesting an increased oxygen consumption caused by DOG-administration at this time point. This is in agreement with the measurements of Schulz et al. [4] who reported an increased oxygen consumption up to the age of 48 hours. With progressing age, the expression of the genes of the Oxphos pathway in glycolytically impaired worms is more and more diminished when compared to controls. Eventually, at very old age, that vast majority of the Oxphos genes in these worms is down-regulated, suggesting a lower mitochondrial ATP production. Because the DOG-treated worms can not produce ATP through the glycolytic pathway, it can only be concluded that the treated worms, after some days, acquire the ability to get along with a lower total energy consumption. Another result that speaks for turned-down overall transcriptional activity under DOG-treatment is that genes belonging to the GO category “RNA polymerase activity” (GO:0034062) are over-represented in a cluster that is up-regulated with age in controls but down-regulated with age under impaired glycolysis (Table S4). The assumption of overall diminished energy consumption and reduced translational activity after the age of one day is supported by the constant down-regulation of *cyp-13A5*, a member of the cytochrome P450 superfamily, in the DOG treated worms. Impairment of *cyp-13A5* is known to be connected with decreased or slow growth [30], [31], a situation that is typical for caloric restriction and longevity. Further hints how the worms might be able to maintain a functioning metabolism with fewer resources will be given below, when network properties of DOG-treated and untreated worms are compared.

Aiming at a reduction of ROS levels, diminishing the production rate of ROS is not the only possibility. An alternative way to protect the cells from oxidative damage is the elevation of the concentration of ROS scavengers like SOD, CTL, peroxiredoxin or glutathione peroxidase (GPX). Schulz et al. measured the activities of these enzymes under glucose-impaired conditions and compared them to untreated controls [4]. They found no increased SOD or GPX activity at any time point evaluated (2 hours, 1 day, 2 days, 6 days) but a significant rise of CTL activity in the glycolytically impaired worms after 6 days. On the transcript level, we neither detect a significant increase of SOD or CTL at any age level (Figure 4), but a significant increase of two GPX (*F26E4.12*, *R05H10.5*; Figure S1) at very old age (20 days). As a matter of course, the discrepancies are not surprising because expression levels do not necessarily reflect enzyme activities and vice versa [32]. The elevation of the GPX at very old age on the transcript level suggests a late impact of the life-extending drug, consistent with the experiments made by Schulz and colleagues. These late changes are attended by continuous, life-long changes in DOG-treated worms when compared to controls, as revealed by the analysis of genes that change from monotonically down- to monotonically up-regulated or vice versa. The *daf-21* gene rises monotonically over the whole life span in DOG-treated worms, in contrast to untreated worms where it is continuously declining over life time. This is in agreement with the fact that a down-regulation of *daf-21* results in reduced life span of *C. elegans* [19], [33]. Unexpectedly, the peroxiredoxin *prdx-2*, a hydrogen peroxide scavenger, is up-regulated with age in untreated worms while it is down-regulated with age in glycolytically impaired worms. This counter-intuitive result might be a consequence of the supposed lower gross energy consumption when monitored over the whole life span, in agreement with the down-regulation of the Oxphos pathway and the assumed long-term reduction of mitochondrial ATP production (Figure 3).

The results of the identification of genes whose expression profiles were negatively correlated between treated and untreated worms, support the conjecture that DOG causes a reduction of the gross level of transcriptional activity with age. Of the 246 genes which are negatively correlated between the two conditions, 154 genes in 5 clusters are down-regulated with age under DOG in contrast to controls (Figure S3). One of the down-regulated clusters is enriched with genes belonging to the gene ontology term “RNA polymerase activity” (GO:0034062), also supporting the above conjecture. Only one cluster containing 22 genes shows the opposite behaviour. This cluster is enriched with genes connected with the regulation of growth (GO:0040008, Table S4).

We have found several hints suggesting that overall energy consumption and gross transcriptional activity are turned down under DOG-treatment when compared to controls. This raises two questions: a) how are the worms able to maintain normal functionality under such conditions and b) why are the worms not using this low energy regime by default, considering the prolonged life span it provides. The answer to the latter question seems to be more obvious: glucose provides an easy available, energy-rich and easy-digestible nutrient. It would be a waste of resources to abstain from natural sugars. Quick access to high-energy nutrients is certainly an advantage in the course of evolution, and is of much greater importance as a long lifetime. Furthermore, it was shown that DOG-treated worms show reduced fertility and a diminished progeny production [4], a fact that certainly also constitutes a competitive disadvantage in natural selection. The above arguments are strengthened by the investigations of Jenkins et al. who demonstrate that *daf-2* mediated life span extension is attended by dramatically reduced fitness [34].

When investigating correlation networks, we discovered clues that might be able to answer the first question as well. The results obtained by the analysis of the difference networks reveal that impaired glycolysis on average increases the global interconnectedness of the genes, supported both by the number of nodes and edges in the DOG-based correlation network, and by the higher mean and maximum node degree in the DOG-network when compared to the DMSO-network. This becomes especially clear when having a look at the Oxphos pathway, found to be one of the most vigorously changed KEGG pathways by impaired glycolysis. We have seen above that the majority of the genes belonging to this pathway are down-regulated at older age in DOG-treated worms compared to controls. Strikingly, the difference network (Figure 5) shows that this down-regulation is accompanied by an improved connectivity in this pathway. Literally, the co-expression of all genes in this pathway is improved when DOG is administrated. This is in agreement with the findings obtained by the calculation of MI, which confirmed that the connectivity of the Oxphos pathway is improved under DOG-treatment (Figure 6, red bars). We can therefore speculate that a better connectivity under impaired glycolysis enables the cell to survive with fewer resources. However, at the same time, the higher connectivity might increase the vulnerability of the network against external perturbations, a characteristic of so called exponential networks [35]. The reason for the increased vulnerability of exponential networks is that - in contrast to scale-free networks - a failure in any node affects many neighboring nodes simultaneously. Therefore, it can be suspected, that prolongation of life span by impaired glycolysis can preferably be achieved under laboratory conditions but not in the wild, another argument that speaks against the usefulness of the glucose-restricted regime as a default metabolic regime.

It is not surprising that co-expression in the glycolysis pathway (Figure 6, green bars) decreases in the case of inhibition by DOG. This might be the consequence of an adaptive response taking place in the cells under impaired glycolysis, where more resources are directed towards oxidative phosphorylation and other processes when energy production by means of glycolysis is inoperable caused by DOG-feeding. Surprisingly, the connectivity of the glutathione pathway is also reduced in the DOG-case, a finding that seems to be a contradiction to the detected longevity of DOG-treated worms. However, we pointed out that (transiently) increased ROS levels can very well be connected with longevity [4], [5].

The perhaps most intriguing structural modification induced by DOG-treatment is the re-organisation of the hubs under DOG-treatment observed in the mTOR pathway. To exemplify this, we can have a look at the *C. elegans* gene *let-363*, the ortholog to the mammalian *FRAP1* gene. The gene encodes the mTOR kinase, a major sensor of cellular energy levels and stress. When ATP or nutrient concentration is high and stress levels are low, mTOR up-regulates translation and stimulates growth and reproduction. Under harsh conditions, *FRAP1* is regulated down, rates of translation are reduced [24], and the cell undergoes a shift towards cell protection and maintenance [36], eventually leading to a prolonged life span [37]. As revealed by our RNA-seq data, the absolute level of expression of *let-363* indeed is decreased at early ages in the long-lived DOG-treated worms (Figure S5), in accordance with the above statements. However, in addition to down-regulation of the expression level, DOG-treatment also promotes *let-363* to a hub (Figure S4), i.e. the gene gains a number of new network connections when DOG is administrated while only being loosely connected in controls. The latter points to the need to put even more emphasis on the investigation of the gene’s interactions in future research, rather than merely exploring their individual levels of expression.

To summarize, we have identified a number of genes and pathways that are potentially involved in the DOG-driven extension of life span of the roundworm *C. elegans*, some of them so far not mentioned in this context. Furthermore, we have shown how the network structure and connectivity of ageing-relevant signalling pathways is re-organised under impaired glycolysis.

## Materials and Methods

### Experimental Design

This study comprises 22 samples: triplicates for controls and treated samples at the age of 1, 5 and 10 days; duplicates for controls and treated samples at the age of 20 days. Each sample involves a number of worms in order to gain a sufficient amount of total RNA for the sequencing process. The exact number of worms per sample varies between several thousands (1 day) and hundreds (20 day). One sample for the 10-day old controls (JA19) had to be discarded because of its high variation between replicates. Additionally, this sample had a low RNA integrity number (RIN) of 6.6 while the mean value for all other samples was about 7.7 with a standard deviation of 0.67.

### *C. elegans* Strains and Maintenance

The *C. elegans* strain used was wild-type N2 (var. Bristol) and was provided by the Caenorhabditis Genetics Center (Univ. of Minnesota). Maintenance was performed as previously published [5], [15].

### DOG-treatment

Treatment of *C. elegans* was carried out on NGM agar plates at 20°C, containing either DOG at final concentration of 5 mM or same volume water as solvent control. As food source for compound treatment heat inactivated bacteria were used. To obtain heat inactivated bacteria, an overnight liquid culture of *E. coli* was treated 30 min at 65°C. The bacteria suspension was then concentrated 20-fold by centrifugation (30 min at 3,200×g) and resuspended in S-buffer containing 10 mM MgSO4 and 5 mg/ml cholesterol.

After L4 larvae stage, worms were transferred to NGM agar plates containing either DOG or water. Nematodes were washed off the plates every 24 hours with S-Basal and allowed to settle by gravitation to remove offspring. The worm pellets were transferred to freshly prepared DOG and control plates and were incubated for indicated time periods.

### Extraction of Total RNA from *C. elegans*

Total RNA was isolated using QIAzol (Qiagen, Hilden, Germany) based on the phenol/chloroform extraction method. Afterwards the RNA was quantified photometrically with a NanoDrop 1000 (PeqLab, Erlangen, Germany) and stored at −80°C until use.

### Transcriptome Profiling Using Deep Sequencing

Agilent Bioanalyzer 2100 and RNA 6000 Nano Kit (Agilent Technologies) was used to check RNA quality in terms of degradation. The RNA integrity number (RIN) was 7.7 in average. Around 2.5 µg of total RNA was used for indexed library preparation using Illumina’s TruSeq™ RNA Sample Prep Kit v2 (order # RS-122-200x) following the manufacturer’s instruction. Libraries were pooled and sequenced (4 samples per lane) using a HiSeq2000 (Illumina) [38] in single read mode with 50 cycles using sequencing chemistry v2. Sequencing ends up with an average of around 50 million reads per sample with a length of 50 bp. Reads were extracted in FASTQ format using CASAVA v1.8.2 (Illumina). The data discussed in this publication have been deposited in NCBI€s Gene Expression Omnibus and are accessible through GEO Series accession number GSE46051 (DMSO controls) and GSE46344 (DOG-treatment).

### RNA-seq Data Analysis

Raw data from the sequencing machine was received in FASTQ format. Read mapping was performed using Tophat [39] and the *C. elegans* reference genome (Ensembl release WS220). The resulting SAM alignment files were processed using the HTSeq Python framework (http://www-huber.embl.de/users/anders/HTSeq; htseq-count in mode ‘union’) and the respective GTF gene annotation, obtained from the Ensembl database [40], in order to assign the sequence reads to the annotated transcripts. Gene counts were further processed using the R programming language [41]. After discarding the genes which solely displayed zero-valued read counts at all age levels, we had available age-dependent expression values for 26,732 genes in the DMSO-case (controls) and 26,569 genes in the DOG-case, respectively. Taken together, complete transcriptome data for 21 samples were available, comprising four different age levels (see Table S1). The read counts were normalized with respect to the size of the individual genes and to the total amount of data obtained in the sequencing process, resulting in an RPKM (Reads Per Kilobase of exon model per Million mapped reads) value for each gene at each time-point [42]. The RPKM values are approximately proportional to the expression levels of the corresponding genes or, more precisely, the ratio of the magnitudes of the RPKM values of a given gene pair reflects the relative abundance of RNA molecules of this gene pair in the sample [42].

We created multi-dimensional scaling (MDS) plots in order to validate the relation of samples and conditions (Figure 1). This plot illustrates dissimilarities between high-dimensional data-sets - here between expression profiles - by finding a set of points in a plane in such a way that the two-dimensional distances between these points reflect the dissimilarities between the datasets. MDS plots were constructed using the cmdscale (classical metric multidimensional scaling) function of the R programming language. The calculations were carried out using default parameters. As dissimilarity measure between samples and , we use , where stands for the Pearson correlation coefficient across all genes, between the samples and . Using this distance measure, highly correlated samples are considered to be close to each other while sample pairs with a mutual correlation close to minus one are considered to be most distant.

### Differentially Expressed Genes

We infer differentially expressed genes (DEG) for each time point separately in order to gain insight into the dynamics of differential expression throughout the whole life span of *C. elegans*, i.e. we observe how many up- or down-regulated genes exist between the two conditions at each time point. The unnormalized gene counts for all genes included in the annotation file were used, however genes were excluded if they had low count values (<100) in one of the samples, which resulted in remaining a set of 16,128 genes. DEG between controls and samples treated with DOG were identified using the edgeR [43] and the DESeq [44] software available within the R statistical language suite [41]. Both packages provide tests for determining differential expression in digital gene expression data using a model based on the negative binomial distribution. Calculated p-values were adjusted for multiple testing using the Benjamini-Hochberg correction algorithm [45]. Genes were regarded as differentially expressed when significance on 99% confidence level was confirmed by both tools. The number of genes which passed this test are listed in Table 1.

### Clustering of Expression Profiles

Genes were clustered according to their temporal profiles using a fuzzy c-means algorithm. We used the function cmeans from the package e1071 of the R programming language. Parameters were defined as: , , . The number of trials for the fuzzy algorithm was set to 30. The optimum number of clusters was determined using a combination of several cluster validation indexes as described by Guthke et al. [20].

### Monotony Test and Monte-Carlo-based Correlation Test

In order to identify genes that change their expression levels monotonically with age, we calculated the Spearman correlation coefficient of each gene’s temporal profile with the linearly increasing curve . In order to incorporate the replicates at each time point, we repeated the calculations by randomly sampling over the replicates at each time point, and by calculating an average correlation coefficient from the re-sampled curves afterwards. We used the calculated correlation coefficient of gene with the linear increasing curve as a criterion to split the genes into the following three groups: if was below −0.75, we considered a gene to be monotonically decreasing with age, if was above 0.75 the gene was considered to be monotonically increasing with age, and if was in between these values, the expression of the corresponding gene was considered non-uniformly. Choosing ±0.75 as a threshold seems to be overly generous, however, given the small number of replicates in connection with the considerable variation between them, the value seems to be reasonable.

We used a permutation approach in order to estimate the significance of the correlation between all pairs of temporal gene expression profiles. We generated a null-distribution of a test statistic by repeatedly permutating the chronological order of the expression values of one gene in the pair, and by subsequently calculating the test statistic for this particular arrangement. To obtain the results at hand, we used the Pearson correlation coefficient as a test statistic, and we carried out 1,000 iterations in order to generate the null-distribution for a given gene pair. For each iteration, we randomly picked one of the replicates at every age level. A one-sided p-value was then calculated by counting the portion of samples whose calculated correlation is at least as extreme as the observed correlation, the latter being calculated using averaged expression values at each time point. Genes were considered significantly negatively correlated if their correlation coefficient was negative, and the p-value, calculated using the permutation approach, was lower than or equal to 0.05.

### Correlation and Mutual Information Networks

The time series of every gene with non-zero RPKM expression values have been used for the estimation of pairwise correlation resulting in two undirected, weighted networks of co-expressed genes, one for each condition. In each network, nodes represent genes, while the correlation between two genes and determines the weight of the edges. Instead of using a fixed correlation threshold, a p-value cutoff of 0.05 was used, estimated using the cor.test function in R. The replicates were included by random sampling. Genes with high variance of expression tend to result in larger changes of correlation and thus were excluded if the p-value was below the selected cutoff. For the first 3 age groups, 3 replicates have been available, while for the last age group (20 days) only 2 are at hand, which results in 3*3*3*2 = 54 possible permutations choosing an temporal profile for one treatment. Accordingly, for each gene there are 54*54−2,916 permutations possible. In order to keep computation time feasible, only 100 out of the 2,916 possible permutations were chosen randomly. Like other biological or social networks, the generated correlation networks are sparsely connected and are supposed to exhibit scale-freeness. This was modelled using linear regression, which resulted in different power law exponents for both of the correlation networks. The parameter characterises the frequency distribution of node degrees in the networks. For the estimation of the difference networks, the fully connected correlation networks were subtracted. The weights of the edges between gene and gene were calculated as the difference of the correlation coefficients , which means that we weight positive and negative correlation equally by focusing on absolute correlation changes. For the subsequent determination of KEGG pathways and GO terms that are mostly affected by the impaired glycolysis, the difference network was pruned using the criterion , which resulted in a sparsely connected network only showing edges connecting gene pairs with substantially changed correlation by DOG. For the pathway investigation, we extracted sub-nets from the global difference network containing the genes of individual GO terms and KEGG pathways, respectively. We considered the 2,881 GO-terms containing more than 3 genes, and all 125 annotated KEGG pathways for *C. elegans*. For these sub-networks, we calculated the fraction of connected genes, and compared this number to the fraction of connected genes in the global difference network. KEGG pathways and GO terms with a significantly increased fraction of connections were determined using Fisher’s Exact Test.

We calculated the mutual information (MI) matrices based on the gene expression profiles utilizing the minet package of the R statistical language suite [12]. MI is an entropy-based measure of the dependency of variables which can be seen as a generalization of correlation. MI-networks (MIN’s) were then constructed from the MI-matrices using the Aracne algorithm [46], a method which uses an information theoretical approach to remove those entries from the MI-matrix which are merely caused by indirect interactions between genes. The standard parameters suggested by the minet package were not changed. As distance estimator in build.mim, we use the Spearman correlation coefficient. In order to analyse the network structure, we utilized the igraph package of the R programming language. When constructing the required igraph object, graphs were declared to be undirected and weighted. MIN’s were created for each of the pathways listed in Table 4 and for 1,000 genes randomly chosen from the whole data-set (background). For simplicity and for the sake of keeping computation time feasible, we averaged over replicates at each time point, thus restricting the calculation to mean values.

For all MIN’s the following parameters have been calculated utilizing the igraph package: a) number of edges, b) average node degree, c) transitivity (often called “clustering coefficient”), d) average coreness, e) average nearest neighbor degree, f) average graph strength, g) average neighborhood size (of 2nd order), h) graph cohesion (“vertex connectivity”), and i) diameter (average length of the shortest paths between any two nodes). A description of these parameters can be found in the review paper by Barabasi and Oltvai [47], in the reference manual for the igraph package, or in the references cited therein. Loosely spoken, the network is more tightly connected if parameters a) through h) are big, and i) is small.

## Supporting Information

Figure S1 **The temporal expression profiles of several anti-oxidant genes.** The plot shows the mean RPKM values as well as the standard deviation versus age in days for five genes encoding superoxide dismutases, three genes encoding catalases and two genes encoding glutathione peroxidases in *C. elegans*. Black curve: controls; red curve: DOG-treatment.
(TIFF)

Figure S2 **Heatmap showing change in gene expression during DOG-treatment for** “**glutathione metabolism” genes.** All genes belong to the KEGG pathway “glutathione metabolism” (KEGG-ID cel00480). The genes are grouped according to the hierarchical clustering of their log2 fold-change profiles (dendrogram on the left side). While most of the genes show down-regulation (colored in red) during DOG-treatment, a group of genes that encodes glutathione transferases is up-regulated (colored in green).
(TIFF)

Figure S3 **Fuzzy c-mean clustering result of genes which are negatively correlated.** 246 significantly negatively correlated over age were identified using a permutation approach. Fuzzy c-mean clustering resulted in a optimal arrangement of 9 clusters (see local peak in CVI plot). Each sub-panel of the figure displays one cluster, showing the mean temporal gene expression profiles of the untreated worms on the left side (red dots), and the corresponding gene expression profiles for DOG-treated worms on the right side (blue rectangles). The lines denote the standard deviation. It can clearly be seen that the profiles belonging to the untreated worms display opposite behaviour when compared to the profiles of the DOG-treated worms. The red-framed clusters, comprising 154 genes in total, contain genes which are up-regulated with age in controls but down-regulated under DOG, respectively. The green-framed sub-panel contains a cluster with 22 genes exhibiting exactly the reversed behaviour. The remaining three clusters (numbers 1, 8, and 9) exhibit irregular but likewise opposite temporal behaviour between treatment and controls.
(TIFF)

Figure S4 **MI-network for genes of the mTOR pathway.** Genes of the mTOR pathway that win or loose hub status when standard diet is changed to DOG feeding. A similar behaviour was not observed in other pathways investigated in this study. Red colored genes are more loosely connected in controls but become a hub in the DOG-network. The opposite behaviour can be observed for the two genes colored in green, which lose their hub status during DOG-treatment.
(TIFF)

Figure S5 **Expression profiles for hub-genes of the mTOR pathway.** Expression profiles for genes of the mTOR pathway that win or loose hub status when standard diet is changed to DOG-feeding.
(TIFF)

Table S1 **RNA-seq sample overview and mapping statistic.** Excel file with RNA-seq sample names, total number of reads and Tophat mapping details.
(XLS)

Table S2 **Constantly up- or down-regulated genes.** Excel file containing significantly differentially expressed genes, which are constantly up- or down-regulated at all four age levels. Expression values are the mean of the RPKM values from the single replicates.
(XLS)

Table S3 **Genes with opposite monotonic behaviour of expression during DOG-treatment.** Excel file containing genes with altered monotonic behaviour during DOG-treatment, including mean RPKM expression values and Spearman correlation coefficients.
(XLS)

Table S4 **Fuzzy c-means clustering result of negatively correlated genes.** The Excel file includes genes with significantly negative correlation between controls and DOG-treatment. The genes are sorted according to the fuzzy c-means clustering result, which yielded 9 clusters. The gene enrichment results were obtained using the DAVID online tool for two selected gene sets (Figure S3).
(XLS)

Table S5 **Pathways and GO terms with disproportionately many connected genes in the difference network.** Excel file containing KEGG-categories and GO-terms which are enriched with connected genes in the correlation based difference network. For each pathway or category the total number of genes, the number of connected genes, and the results of the statistical test are given.
(XLS)

Table S6 **Mutual information network parameters.** Excel file containing the estimated parameter values from the different mutual information networks, shown in Figure 6.
(XLS)

Table S7 **DEG analysis results by DESeq and edgeR.** Excel file containing the results from the analysis of DEG identified by DESeq [44] and edgeR [43] for each of the 4 age groups. The table contains the mean RPKM expression values, fold-changes, and statistical test results.
(XLS)
