# Supplementary figures and images for: Extension of Life Span by Impaired Glucose Metabolism in Caenorhabditis elegans Is Accompanied by Structural Rearrangements of the Transcriptomic Network
Source: PLoS One. 2013 Oct 30;8(10):e77776. doi: 10.1371/journal.pone.0077776 (PMC3813781; doi:10.1371/journal.pone.0077776)

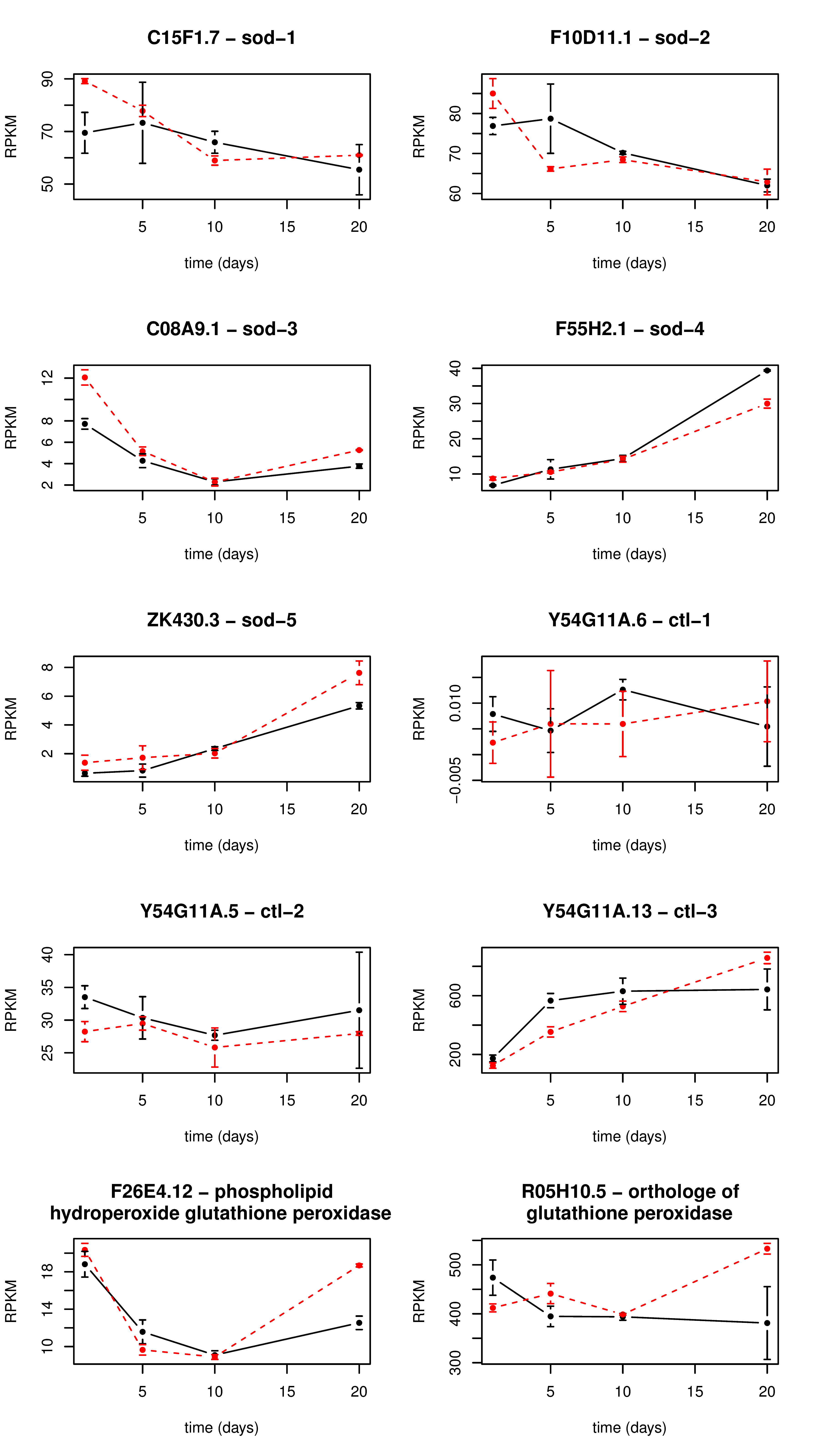

Supplement: Figure S1 — The temporal expression profiles of several anti-oxidant genes. The plot shows the mean RPKM values as well as the standard deviation versus age in days for five genes encoding superoxide dismutases, three genes encoding catalases and two genes encoding glutathione peroxidases in C. elegans. Black curve: controls; red curve: DOG-treatment. (TIFF) [file pone.0077776.s001.tiff]

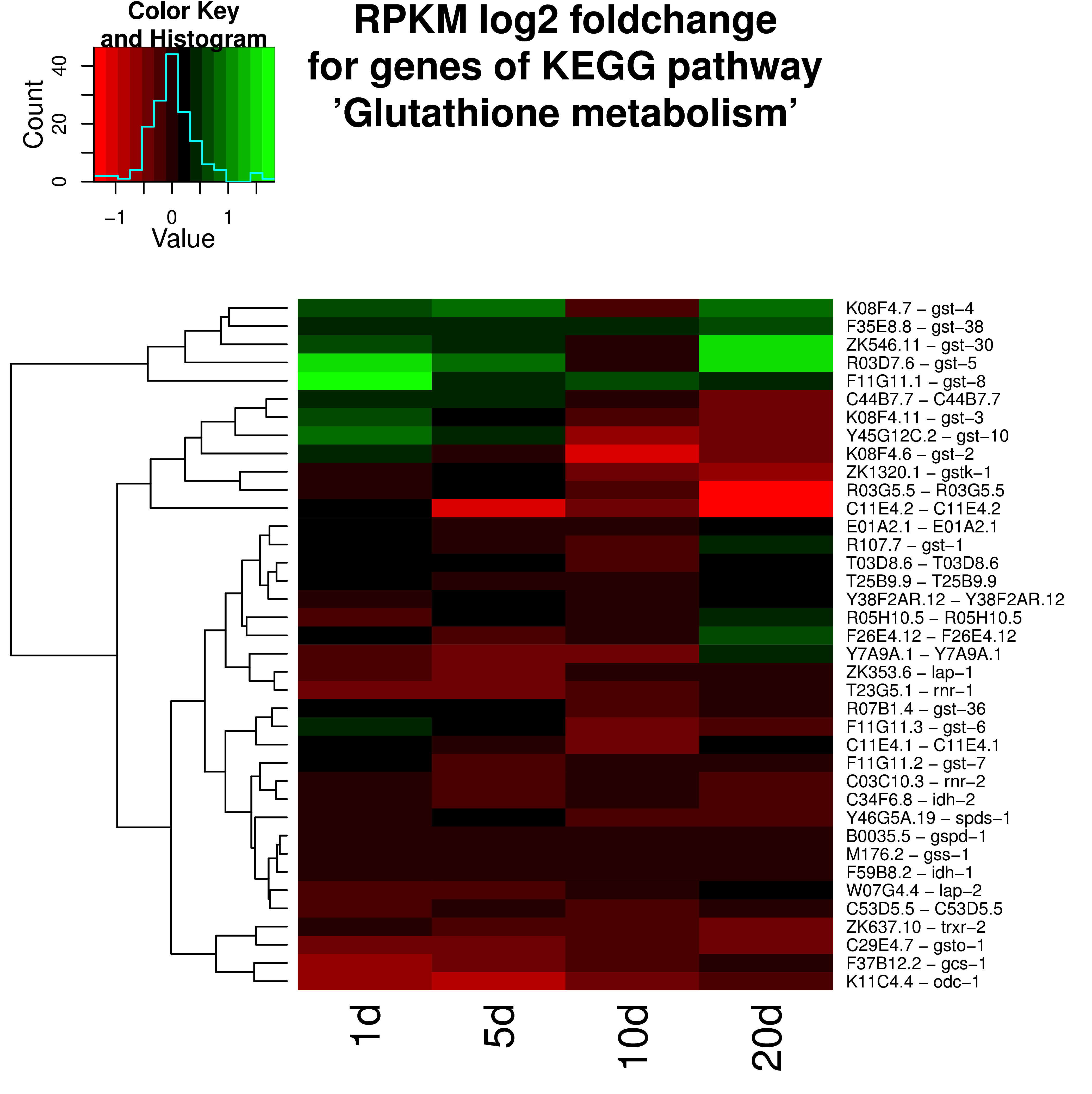

Supplement: Figure S2 — Heatmap showing change in gene expression during DOG-treatment for “glutathione metabolism” genes. All genes belong to the KEGG pathway “glutathione metabolism” (KEGG-ID cel00480). The genes are grouped according to the hierarchical clustering of their log2 fold-change profiles (dendrogram on the left side). While most of the genes show down-regulation (colored in red) during DOG-treatment, a group of genes that encodes glutathione transferases is up-regulated (colored in green). (TIFF) [file pone.0077776.s002.tiff]

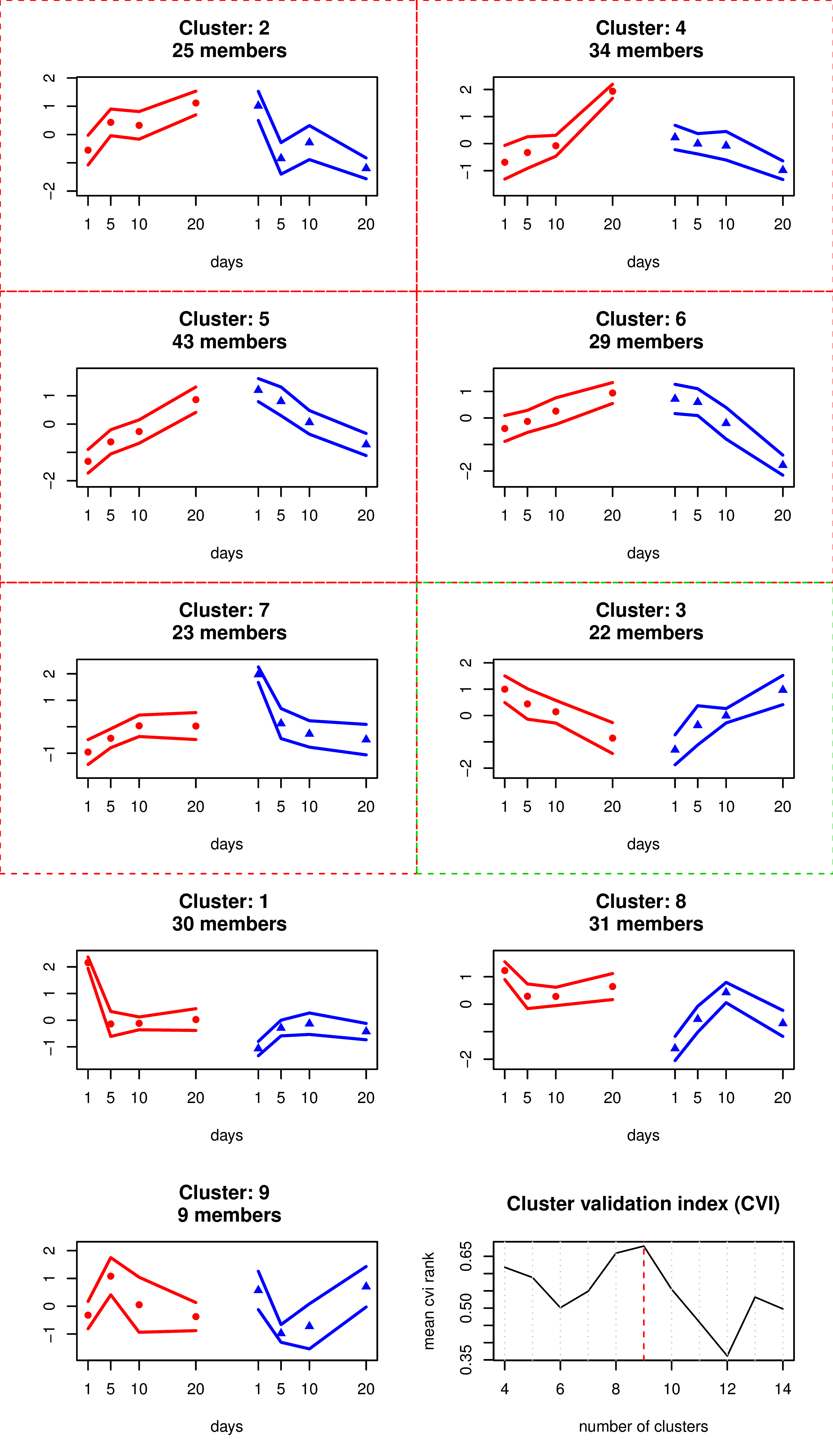

Supplement: Figure S3 — Fuzzy c-mean clustering result of genes which are negatively correlated. 246 significantly negatively correlated over age were identified using a permutation approach. Fuzzy c-mean clustering resulted in a optimal arrangement of 9 clusters (see local peak in CVI plot). Each sub-panel of the figure displays one cluster, showing the mean temporal gene expression profiles of the untreated worms on the left side (red dots), and the corresponding gene expression profiles for DOG-treated worms on the right side (blue rectangles). The lines denote the standard deviation. It can clearly be seen that the profiles belonging to the untreated worms display opposite behaviour when compared to the profiles of the DOG-treated worms. The red-framed clusters, comprising 154 genes in total, contain genes which are up-regulated with age in controls but down-regulated under DOG, respectively. The green-framed sub-panel contains a cluster with 22 genes exhibiting exactly the reversed behaviour. The remaining three clusters (numbers 1, 8, and 9) exhibit irregular but likewise opposite temporal behaviour between treatment and controls. (TIFF) [file pone.0077776.s003.tiff]

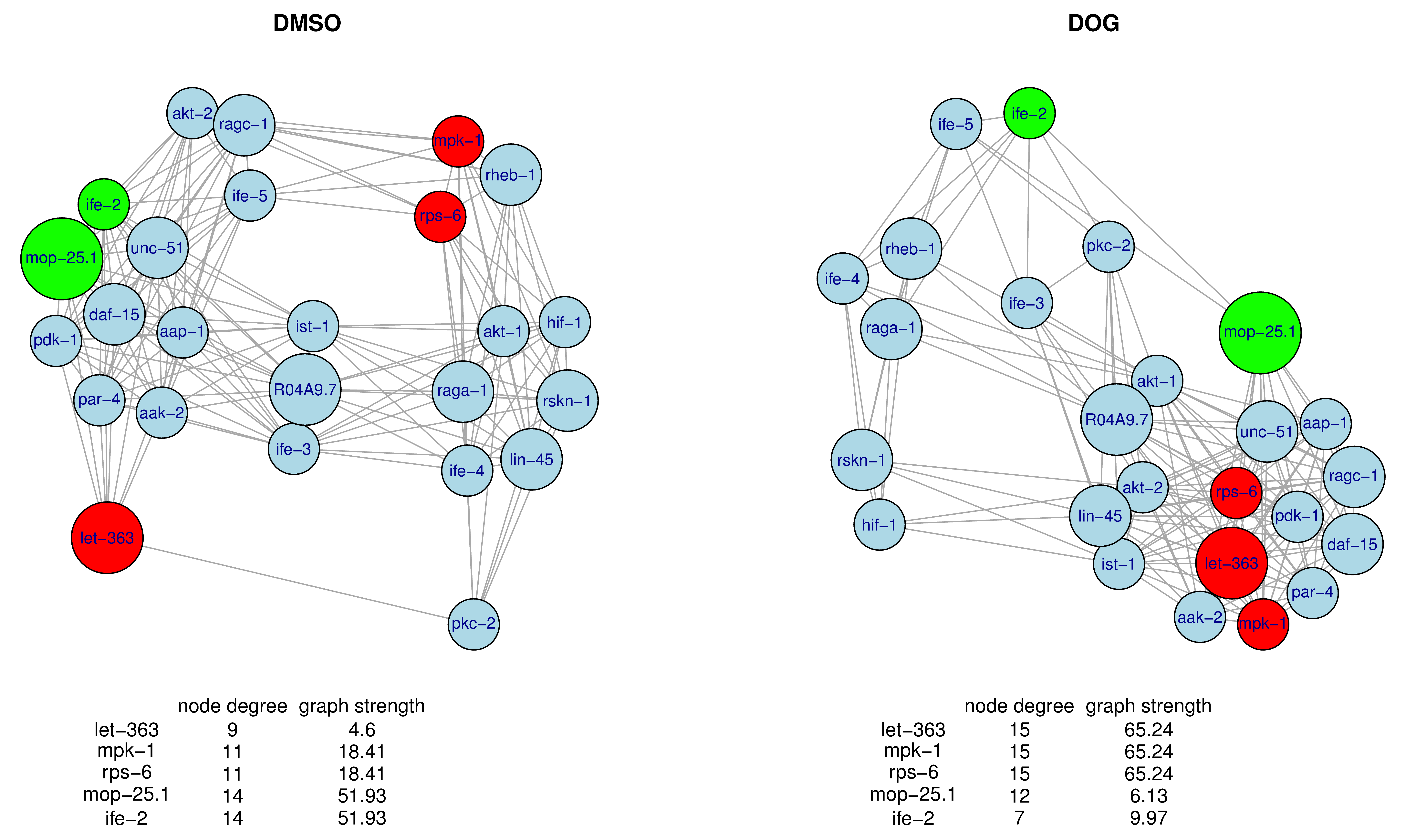

Supplement: Figure S4 — MI-network for genes of the mTOR pathway. Genes of the mTOR pathway that win or loose hub status when standard diet is changed to DOG feeding. A similar behaviour was not observed in other pathways investigated in this study. Red colored genes are more loosely connected in controls but become a hub in the DOG-network. The opposite behaviour can be observed for the two genes colored in green, which lose their hub status during DOG-treatment. (TIFF) [file pone.0077776.s004.tiff]

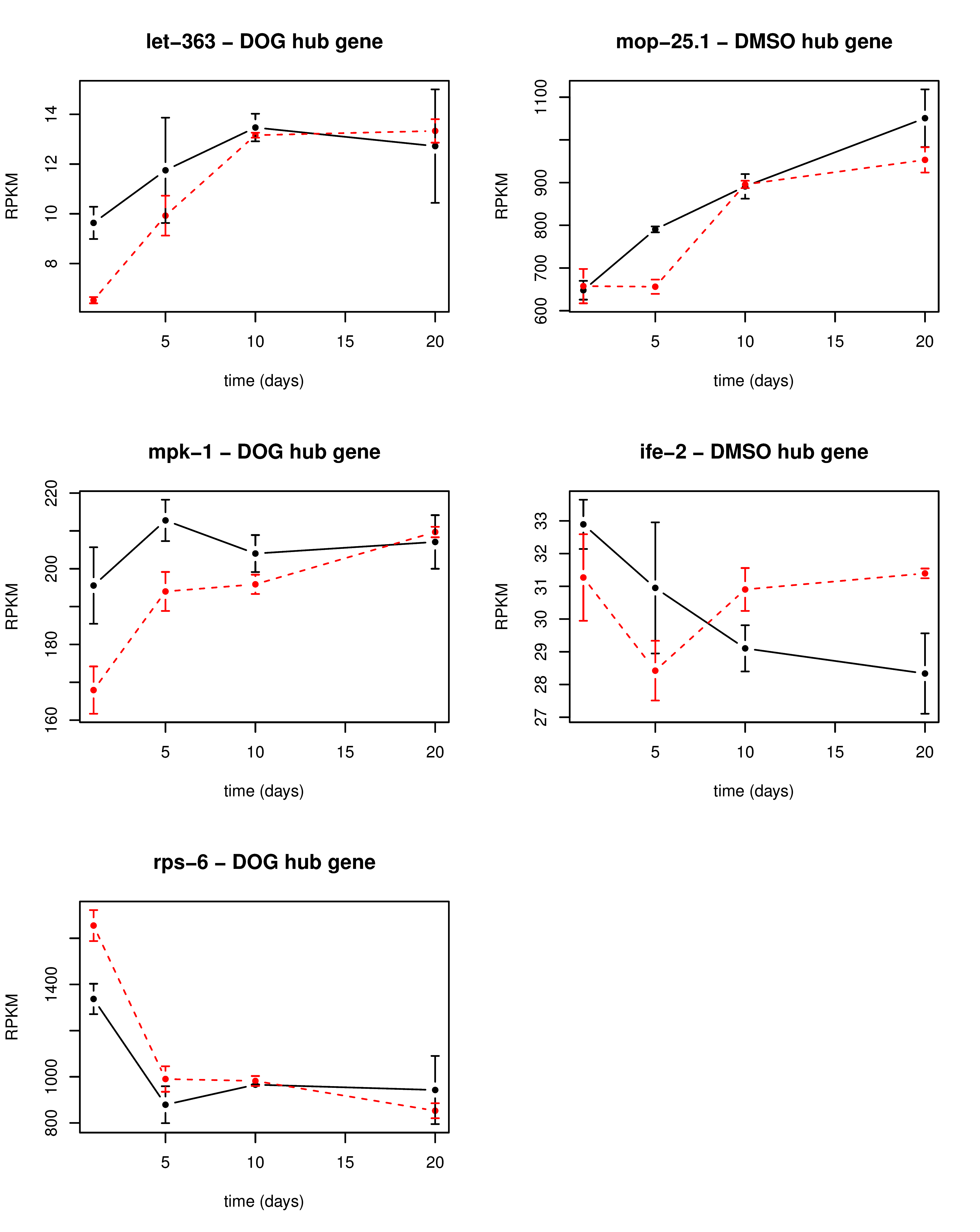

Supplement: Figure S5 — Expression profiles for hub-genes of the mTOR pathway. Expression profiles for genes of the mTOR pathway that win or loose hub status when standard diet is changed to DOG-feeding. (TIFF) [file pone.0077776.s005.tiff]
